# Social Cohesion and COVID-19: Integrative Review

**DOI:** 10.2196/51214

**Published:** 2024-11-21

**Authors:** Paul Ware

**Affiliations:** 1 Department of Social and Community Health School of Population Health University of Auckland Auckland New Zealand

**Keywords:** social cohesion, social capital, COVID-19, infrastructure, tool, social, economic, interpersonal, interpersonal relationship, emotions, pandemic, engagement, health behaviors, resilience, emotional well-being, well-being

## Abstract

**Background:**

Nations of considerable wealth and sophisticated health care infrastructures have experienced high rates of illness and death from COVID-19. Others with limited economic means and less developed health systems have achieved much lower burdens. To build a full understanding, an appraisal of the contribution of social relationships is necessary. Social cohesion represents a promising conceptual tool.

**Objective:**

This study aimed to examine scholarship on social cohesion during the COVID-19 pandemic: specifically, the constructions of social cohesion being deployed, the variables chosen for representation, and the effects of and on social cohesion being reported.

**Methods:**

The PubMed, Scopus, and JSTOR databases were searched for relevant journal articles and gray literature. A total of 100 studies met the inclusion criteria. Data were extracted and analyzed from these using spreadsheet software.

**Results:**

Several constructions of social cohesion were found. These concerned interpersonal relationships, sameness and difference, collective action, perceptions or emotions of group members, structures and institutions of governance, locally or culturally specific versions, and hybrid or multidimensional models. Social cohesion was reported to be influential on health outcomes, health behaviors, resilience, and emotional well-being, but there was some potential for it to drive undesirable outcomes. Scholarship reported increases or decreases in quantitative measures of social cohesion, a temporary “rally round the flag” effect early in the pandemic, the variable impacts of policy on social cohesion, and changing interpersonal relationships due to the pandemic conditions. There are numerous issues with the literature that reflect the well-documented limitations of popular versions of the concept.

**Conclusions:**

Social cohesion has been used to express a range of different aspects of relationships during the pandemic. It is claimed to promote better health outcomes, more engagement with positive health behaviors, and greater resilience and emotional well-being. The literature presents a range of ways in which it has been altered by the pandemic conditions. There are significant weaknesses to this body of knowledge that greatly impede its overall quality.

## Introduction

### Overview

The COVID-19 pandemic has offered a challenge to those seeking to understand the operation of social relationships and patterns of health and illness. Throughout what is likely the first truly global pandemic to begin in the information age, humans have had access to real-time data from across the globe. What we have seen has frequently surprised us. For example, the 2019 Global Health Security Index positioned the United States and the United Kingdom in the top 2 positions in the league table of pandemic preparedness [1], yet these nations have experienced among the highest rates of illness and death from COVID-19 on the planet [2]. The same report placed, for example, Aotearoa–New Zealand (Aotearoa-NZ) and Singapore much lower in the rankings, but these nations have managed to limit their burdens of this kind [2].

Analyses have sought to explain unequal COVID-19 outcomes between populations using, among other independent variables, features of physical geography [3], previous experiences of infectious disease outbreaks [4], ethnic composition [5], and even leaders’ gender [6]. Although some of these may make important and valid points, none tell the whole story: an understanding of the patterns of health and illness cannot be complete without attention to social determinants. After all, it is physical, in-person interaction between humans that allows the virus to spread. In most cases, a nation’s ability to slow or stop the spread of the virus has depended on collective action from a large majority of its population. More fundamentally, the ability of a government to enact policy for protection of public health in the face of COVID-19 is contingent on there being political infrastructure to permit it and, following this, the likelihood that the policy will be adhered to at the population level. At every juncture, the progress of the disease is contingent on social relationships.

### Social Cohesion

One useful tool for the examination of social relationships is social cohesion. Comprehensive accounts of social cohesion have already been written recently [7-9]; however, a brief discussion here may provide context to the current work. Most basically, it is a tool for the analysis or characterization of social relationships. There are several theoretical lineages underpinning social cohesion as it currently appears, their relative presence in different incarnations of the concept being frequently noted in the more critical literature [10-14]. The inception of social cohesion into academic inquiry is usually attributed to Durkheim [15], who sought to understand what brings and holds societies together in the context of the rapid industrialization of the 19th and early 20th centuries. His answers to this question—shared values and culture and functional interdependence—are at the foundation of one theoretical lineage, present through Parsons’ [16] and the functionalists’ scholarship and are a notable feature of more recent work by Jenson [10]. Attention to the Marxian theory of capitalist socioeconomics, the division of labor and wealth inequality is also frequently present, with inequality presented as an impediment to social cohesion, which in such accounts is largely synonymous with order and stability [17].

Some scholars, such as Green and Janmaat [11], indicate the importance of the state in the operation and maintenance of social cohesion and, in doing so, draw attention to the work of political theorists. Liberal, contractarian, romantic conservative, and French republican thought traditions are each cited in this work. Such ideas are carried through into the contemporary social cohesion scholarship from policy, development, and political science fields. In this general area, social cohesion is again a question of a social order, underpinned by particular understandings of the nature of the human condition and sociality, for which the state is ultimately responsible.

Another influential theoretical lineage, though one that has seen rather less explicit acknowledgment in the more recent social cohesion literature, grew from roots in social psychology. The pioneering work by Le Bon [18] on behavioral contagion paved the way for a range of contributions, including those from Freud [19], Lewin [20], and the field theorists and group identity scholars, such as Hogg and Turner [21]. Although this work was primarily small-group experimentation, some of its principles and lexicon have become established in the social cohesion scholarship seeking to characterize larger groups, of course in those from psychological fields making reference to the topic [22], but also notably in the characterization of intergroup relationships, identification with the local or national community, and in the nature and operation of trust.

The more recent construction of social capital concepts adjacent to social cohesion has also been influential. Building in part on the work of social network analysts [23], Bourdieu [24], and Coleman [25] each introduced their own versions of social capital. This concept draws attention specifically to interpersonal relationships and the opportunities and resources these may offer to the individual. In this form it had relevance to social cohesion but still remained reasonably distinct: where social cohesion was, for the most part, to operate at the population level, social capital was concerned with individual-level social networks. However, with the popular version of social capital from Putnam [26-28] the boundaries between the two became less clear. Putnam’s work departed from the use of the individual as the unit of analysis to focus on groups’ and communities’ extent and practices of civic engagement and the operation of social norms, the implications these have for population-wide trust, and, following this, the effectiveness of democracy and successful economic growth. Putnam’s social capital thus began a literature trajectory that appraised variables and conditions that would have found a comfortable home under the social cohesion banner as social capital, and in many cases that treats the two as synonymous (eg, Kawachi and Berkman [29]).

Most recently, policy and development scholars, and, following them, those from several other fields, including public health, economics, and geography, have brought these theoretical lineages of social cohesion and its fraught relationship with social capital into dialog with contemporary social and political problems or change and have sought to operationalize them quantitatively. The manner in which social cohesion is represented in such work is often contingent on the problems or processes of change under scrutiny. For example, scholars seeking to draw attention to the human casualties of the neoliberal dismantling of the welfare state, tend to foreground the problems of inequality [30]; those looking to solve the problem of ethnic intergroup tension frequently look to explanations around shared culture, values, and interpersonal connection [31]; and those concerned about the social order and operation of democracy can commonly be found emphasizing the role of the state and the quality of its institutions [32]. It has been used to represent or understand—among many other sets of relations—collective action [33], relationships between populations and the state [24,34], trust [29], and neighborhood networks’ resilience to hardships or shocks [35].

The diversity of the lineages of social cohesion and contexts in which it has been deployed have produced significant diversity within the concept. In practice, there are many different social cohesions, and there is rarely any basis to claim one of them more valid or useful than the next. This has long been observed in the literature: Bernard [17] famously referred to social cohesion as a “quasi-concept,” imbued with “a vagueness that makes [it] adaptable to various situations...to follow the meanderings and necessities of political action from day to day,” and Cheong et al [36] suggested that it is “a moveable feast, aligned with the political and ideological positions of policy makers, practitioners, and academics.” Despite the currency it has come to hold in both policy and academic debates, it is rare that all parties to a conversation on the topic are talking about the same thing.

Although this ambiguity has produced definitional and conceptual confusion, it may also lend the social cohesion concept a flexibility that can be a source of strength in examining human sociality. Given the collective nature of the problems of COVID-19 and the requirement for collective solutions, it appears to be especially promising in the analysis of social relationships in such a context. This work is an initial step into such a line of inquiry. Beyond this specific topic, and as the direct impacts of the COVID-19 pandemic diminish in magnitude, the accumulation and persistence of further wicked problems, such as environmental degradation and climate change, poverty and inequality, recurring financial-economic crises, etc, the imperative for understanding collective behaviors becomes ever more pressing. Social cohesion is a concept that might be of use to human endeavors therein.

### The Current Work

As part of a broader research project using social cohesion to understand the operation of social relationships within and upon the COVID-19 pandemic, a review of what others have contributed is necessary. Reported here are the processes and results of an integrative review on the topic. Given the definitional ambiguity and broadness of the scope of the social cohesion concept, in addition to its effects, it was of interest to investigate the different ways in which social cohesion has been constructed. It is thus not the intention of this work to seek solutions to its ambiguity and vagueness, or indeed to imprint its own framework or claims as to the nature of social cohesion onto the endeavor. Rather, reported here is an exploratory mission to seek out how social cohesion has been deployed by others: which versions are being used, how they are being used, and the effects on and of the experience of the pandemic being claimed.

## Methods

### Search Strategy

A computerized search strategy was conducted on the PubMed, Scopus, and JSTOR databases on October 10, 2022, then repeated on August 8, 2024. The dual purpose of the review—to explore the range of versions of social cohesion being deployed to characterize the social relationships in operation during the COVID-19 pandemic and the effects thereon and thereof—necessitated the casting of a broad net in the literature search. This need was magnified by the newness of the topic. Given the slippage between social cohesion and such adjacent concepts as social capital, it was worthwhile to maintain singular interest in those accounts specifically and explicitly using the social cohesion concept as an important component in the analysis. The following search string was used: “social cohesion” AND (“coronavirus” OR “Covid”).

Searches were limited to journal articles and gray literature published since 2020 to exclude results from before the COVID-19 pandemic. For the PubMed search, the search parameters were set at title and abstract. For the Scopus search, this was widened to also include keywords, and for the JSTOR search, to include the whole text as initial title and abstract searches alone yielded limited results.

From an initial return of 698 results, 89 (12.75%) were reviewed. A further 11 (1.5%) articles and reports were included from the reference lists, bringing the total number of papers to 100 (14.3%). Inclusion criteria were as follows:

1. Social cohesion formed a substantial part of the analysis. Across social scientific scholarship, the term is frequently used without a definition or significant discussion. Including papers where social cohesion was present in this manner would not be of value to the task of generating detailed knowledge on social cohesion during the COVID-19 pandemic.
2. Discussion was present on COVID-19 outcomes or social cohesion in the context of the pandemic. The purpose of this review being to understand the relationships between COVID-19 and social cohesion meant that any papers not dealing with these were not relevant.
3. The full text was available through the associated database.
4. The full text was available in English.

### Data Extraction

All 100 studies were accessed electronically. In each case, PDF files were downloaded from the database on which they were found. Data were extracted using the integrative method from Whittemore and Knafl [37], which was chosen for its capacity to draw together a range of methodologies. This consists of data reduction and coding, display, comparison, and the drawing of conclusions. A coding matrix was developed using the Microsoft Excel spreadsheet software. During initial coding, the literature was arranged by methodology and discipline. Following this, through an inductive process, it was organized by further categories becoming salient during a second pass. These included the nature of the work (empirical, analytical, or theoretical), the units of analysis, the objects of focus, the construction of social cohesion being deployed, and the outcomes being described. A third pass over the literature informed by this was then conducted, which yielded the thematic structure described in the following section. The extraction focused in particular on the manner in which social cohesion was conceptualized, the components or indicators thereof proposed, and the argument being presented regarding its operation during the pandemic.

## Results

### Selection and Exclusion Process

**Figure 1:**
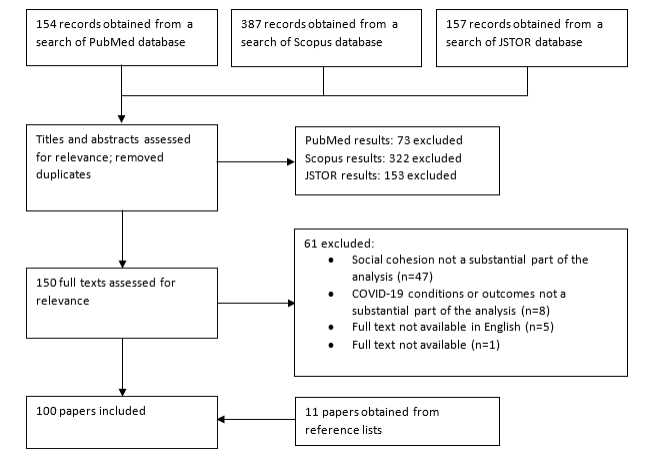
Literature selection and exclusion diagram.

The selection and exclusion process is shown in Figure 1.

### Study Features

Tables 1 to 3 provide details of the papers reviewed relating to location of interest, the methodological approach, and the effects on or of social cohesion being investigated.

**Table 1:** Reviewed papers and their locations of interest (N=100).

Location of interest	Papers, n (%)	References
United Kingdom	13 (13)	[13,38-49]
United States	16 (16)	[50-65]
Germany	6 (6)	[66-71]
Australia	8 (8)	[72-79]
Aotearoa–New Zealand	4 (4)	[80-83]
Iran	3 (3)	[84-86]
South Africa	2 (2)	[87,88]
Indonesia	1 (1)	[89]
Spain	1 (1)	[90]
China	4 (4)	[91-94]
Canada	1 (1)	[95]
Argentina	1 (1)	[96]
Peru	1 (1)	[97]
Denmark	1 (1)	[98]
Kenya	1 (1)	[99]
Italy	2 (2)	[100,101]
Poland	1 (1)	[102]
Romania	1 (1)	[103]
Japan	2 (2)	[104,105]
Chile	1 (1)	[106]
Netherlands	1 (1)	[107]
Brazil	1 (1)	[108]
Colombia	1 (1)	[109]
Switzerland	1 (1)	[110]
Two or more nations	22 (22)	[111-132]
No specific location	4 (4)	[133-136]

**Table 2:** Reviewed papers and their modes of inquiry or analysis (N=100).

Methodology	Papers, n (%)	References
Analytic commentary	16 (16)	[13,50,79,80,84,89,111-118,124,125]
Theoretical commentary	9 (9)	[56,83,88,100,126,127,133-135]
Empirical: quantitative	47 (47)	[39,41,48,49,51,53-55,57,59-62,64-66,68,70,71,73,75-78,81,86,91-95,98,99,101-106, 119,120,123,129-132]
Empirical: qualitative	19 (19)	[38,46,47,52,58,63,72,74,82,85,87,90,97,107-109,121,122,128]
Empirical: mixed methods	5 (5)	[42-45,96]
Theoretical and empirical combined	4 (4)	[40,67,69,110]

**Table 3:** Effect on or of social cohesion under investigation (N=100).

Effect on or of social cohesion being investigated	Papers, n (%)	References
Interplay between pandemic conditions and social cohesion	35 (35)	[13,41-44,46,48,52,57,59,66,68,69,71,74,79,80,82-84,87,88,90,98,101, 102,106,112,113,118,120,126,133,134,136]
Social cohesion as explanatory variable for patterns of health and illness	11 (11)	[39,53,54,56,62,111,119,123,127,129,130]
The role of governance institutions in maintaining social cohesion during the pandemic	9 (9)	[50,94,99,108,114-116,124,125]
Social cohesion as explanatory variable for adherence to public health measures	13 (13)	[38,40,55,58,60,75,78,85,92,93,104,121,122]
Relationship between social cohesion and mental or emotional well-being during the pandemic	14 (14)	[51,61,64,65,73,76,77,81,86,91,95,100,105,132]
Social cohesion as resilience to pandemic hardships	14 (14)	[49,63,67,70,72,89,96,97,103,109,110,117,131,135]
Voluntary work as determinant of social cohesion during the pandemic	2 (2)	[45,47]
Within-group social cohesion as determinant of medical professionals’ performance	2 (2)	[107,128]

### Different Models of Social Cohesion

A range of constructions of social cohesion were presented. These may be sensibly organized as clusters of objects that are used to represent social cohesion one way or another. These clusters commonly describe the same or similar sets of behaviors or situations using different terminology. In total, 7 categories are proposed for the papers reviewed: interpersonal relationships, sameness and difference, groups’ collective action or members’ choosing to act toward the overall benefit of the group, the sum of the perceptions or emotions of group members, and the operation of structures and institutions of governance. The sixth category is a small one that contains locally and culturally specific constructions of social cohesion, and the seventh consists of accounts presenting large hybrid and multidimensional models incorporating several other categories. In each case, the chosen version of social cohesion is represented by particular constructs or variables that act as a representation of the whole. Table 4 contains a summary of this information.

Each model of cohesion naturally implies the units of analysis between which relations are being characterized. The interpersonal group measures characteristics of the individual human, and in its purpose to appraise the operation of their social networks, often resembles what has been more commonly characterized as social capital. The individual is also used as the unit of analysis in the process of calculating the sum of emotions and perceptions. Analyses of sameness and difference tend to span the individual and group. A common example of this practice is the measurement of individual group members’ characteristics to make inferences about the group. The same general practice is also apparent in the working together category. Analysis of institutions and governance usually situates the particular structures under scrutiny as the units of analysis.

**Table 4:** Models of social cohesion and constructs chosen for measurement.

Locus and aspect under analysis		References
**Interpersonal relationships**		
	Provision of social support	[38,48,52,55,57,60,61,63,72,82,90,93,97,109,113,131]
	Interpersonal reciprocity	[53,104]
	Information sharing	[38,72,104]
	Number of social interactions	[39,41,44-46,48,49,56,59,67,89,94,96,120]
	Quality of social connections	[41,45,46,49,55,61,64,73,76-78,84,89,90,92-95,113,117,120,132,133]
	Relative salience of different types of social relationships	[44-46,54,120]
	Within ethnic group	[43,48,72,73,82]
	Within professional group	[107,128]
**Sameness and difference**		
	Interethnic group relations	[63,72,82,106]
	Shared identity	[13,43,44,49,75,78,99,134]
	Norms	[58,91,94,109,134]
	Values	[69,93,95,109,121]
	Behavioral conformity	[38,58,69,72,122]
**Working together for the good of the collective**		
	Collective action	[38,48,50,74,75,87,103,111,114,115,124]
	Cooperation	[49,69,90,99,102,114]
	Collaboration	[88,125]
	Limited conflicts of interest	[117]
	Pursuit of a shared goal	[101,107,109,121]
	Meeting social responsibility	[13,38,64,87,94,108,119,122]
	Orientation to common good	[50,68,85]
	Mutual aid	[84,117]
	Altruism	[50,73]
	Solidarity	[50,91,108,117,118,121,122,135]
**The sum of individual group members’ emotions or perceptions**		
	Feelings of togetherness	[13,43,65,69,74,94,122,131]
	Trust in others in the nation	[44,46,66-68,75,79,90,108,119]
	Trust in those from local community	[39,41,43,44,51,53,57,61,64,67,68,73,75-77,84,85,90,91,95,105,109-111,119,132]
	Trust in others to follow public health rules	[42-44,69]
	Trust in politicians or political institutions	[43-45,48,50,67,69,75,78,79,86,87,90,96,98,99,111,119]
	Perceived cohesion	[41,85]
	Feelings of belonging	[13,43,49,53,60,64,65,67,79,82,86,105,109,110,131]
	Feelings of safety	[65,73,130]
**The operation of institutions and structures of governance**		
	Effectiveness of democracy	[108,123]
	Integrity of the social contract	[116]
	Within-nation inequality	[79,115,116,119]
	Action to uphold human rights	[98,135]
	Strength of state institutions	[108,121,125]
	Integration of state institutions	[85,99,126]
	Management of disorder	[127,130,135]
	Effective leadership	[115,125]
Locally or culturally specific expressions		[50,69,88,90,91,103]
Hybrid or multidimensional models		[13,48,67,69-71,75,82,99,121,129,135]

### The Different Effects of Social Cohesion

#### Health Outcomes

Those works presenting social cohesion as a determinant of health most commonly held it to be producing comparatively positive population health outcomes from COVID-19. Such reporting included the negative association of social cohesion (frequently referring to social capital, either explicitly or implicitly, as a proxy) with the likelihood of death [54,123,129,130]; or the spread of infections of COVID-19 in groups and populations [53,56,127,129,130]; hospitalization [62]; or antibody response to vaccination [39]; or, in the case of some analytical and theoretical work, a less precisely defined and more general “health outcomes” [111]. For example, Gallagher et al [39] used a 5-item questionnaire to measure frequency of contact with neighbors, and perceptions of neighborhood trustworthiness, willingness to help, similarity and friendship; and vaccination-related blood antibody concentration among 676 people from the United Kingdom, finding a positive association between their measure of social cohesion and blood antibody concentration.

Some works sought to isolate the effect of different components or variables within their model of social cohesion on health outcomes from COVID-19 [53,54,119] or different effects of the same components on different populations [53]. For example, Elgar et al [119] drew on existing data from surveys across 84 countries and used a Putnam-inspired construct measuring trust in other people, membership of community groups, civic activity, and confidence in the state (thus resembling social capital) to investigate a hypothesized association between social cohesion and COVID-19 deaths in the early days of the pandemic. They reported that while mortality was positively associated with interpersonal trust and group affiliations, it was negatively associated with civic engagement and confidence in the state. Ransome et al [53] measured feelings of belongingness, trust in neighbors, perceptions of neighbors’ willingness to help, civic and social participation, and collective engagement across a selection of neighborhoods in Philadelphia, looking for any associations with rates of COVID-19 diagnosis. They found that social cohesion operated differently in different places: neighborhoods predominantly occupied by African Americans found some of their indicators of choice to be associated with higher rates of diagnosis, while those in which African Americans were the minority saw the same indicators associated with lower rates.

#### Information for and Practice of Health Behaviors for the Prevention of COVID-19

Several studies described the effects of their chosen construction of social cohesion on the promotion of health behaviors intended to prevent transmission of SARS-CoV-2. These included commentary on those behaviors made compulsory by legislation and access to or transmission of information around them. This body of work includes notable contributions from those who deployed qualitative methods to explore in detail the motivators and barriers for practicing recommended and required public health behaviors [38,60,85,121,122]. Each of these introduced social cohesion at the latter stages of their analyses to draw together a collection of influences on opinions and behaviors into a single explanation. In keeping with the qualitative approach, these presented a more complex arrangement of forces than the single-axis scales or binaries commonly expressed by quantitative work. For example, Zimmerman et al [122] used social cohesion to explain those behaviors informed by feelings of togetherness, commonality, empathy, and compassion, linking this to the spread of uptake of health-promoting behaviors across a group. They noted that these can encourage closer adherence to public health guidelines through care for the collective, while at the same time may cause people to deviate from guidelines and regulations when doing so is perceived to benefit another or others who may be in need.

Others produced quantitative analyses using self-report surveys to capture the practice of specific sets of COVID-19–related health behaviors [55,75,93,96]. For example, Cheng and Lo [55] surveyed older adults from the United States on the number of preventative behaviors from a given list in which they engaged and a 3-item Likert scale measuring social cohesion at the neighborhood level. They reported a positive association between the two. Cárdenas et al [75] surveyed a large sample of Australians to investigate whether engaging in physical distancing and hand hygiene behaviors were susceptible to sociopolitical determinants. They used a model of social cohesion incorporating social identification, confidence in government, and social relations captured by a 14-item tool. They reported a complex set of findings in which their measures relating to social cohesion are spread across being positively associated with these health behaviors, negatively associated with them, evidencing no apparent relationship, or associated with one and not the other.

The place of social cohesion in the distribution, provision, and accessing of good-quality health-related information has also been of interest to scholars [38,72,104]. The interview research by Burton et al [38] and Healey et al [72] presented the sharing of health information as a form of social support and, in turn, as a component of social cohesion. Machida et al [104] suggested that the offering and uptake of health information between groups bound by ethnic and cultural sameness may be an explanation for the positive relationship they found between social cohesion and rates of vaccination.

Some authors engaged in commentary on relationships between social cohesion and whether people choose to become vaccinated against COVID-19 [40,78,92,104]. For example, Machida et al [104] in a cross-sectional study in Japan found social capital (in their Putnam-derived construction containing social cohesion) to be associated with both previous vaccination and intent to receive a booster vaccine.

#### Social Cohesion Promoting Resilience and Emotional Well-Being

The effects of social cohesion on resilience and mental and emotional well-being have seen significant attention in the literature. These works commonly noted the heightened states of stress and anxiety brought about by the pandemic and the isolating effects of the physical distancing measures that were used to prevent transmission of SARS-CoV-2. There are those who referred to the resilience of groups in this respect, proposing that their chosen model of social cohesion is supportive of this [49,59,60,63,64,70,72,76,77,89,96,97,103,107,109,110,117,128,131]. For example, Rela et al [89] offered an analysis of the action of a Putnam-influenced fusion of social cohesion and social capital in Indonesia. An argument is presented for social cohesion as a determinant of community resilience, defined as the capacity of a group to cope with changing environments while continuing to improve the living conditions of its members. Garcia-Rabines and Bencich [97] and Healey et al [72] both argued that within-group cohesion in marginalized communities (transgender women and ethnic minorities, respectively) can act as a source of resilience in times of crisis in the forms of practical and moral social support. Group resilience is also, by some, explicitly held as important for groups’ protection against psychosocial distress and clinical versions of this [67,96]; and groups of health care professionals’ ability to maintain stability and the provision of care [107,128]. Most commonly, however, the various constructions of social cohesion at the group level are offered as a resource for individuals’ protection from poor mental health outcomes [51,65,73,81,86,91,95,100,105,132,135]. For example, Best et al [95] surveyed 1381 Canadians on perceptions of social cohesion in their neighborhoods and levels of panic, depression, emotional stability, and worry, alongside a range of other variables. They found that the COVID-19 pandemic and the restrictive responses to it were responsible for heightened distress, but that their social cohesion indicators were unequivocally negatively associated with all measures thereof. O’Donnell et al [73] reported, based on longitudinal research with Australian informants, that neighborhood-level social cohesion was associated with lower levels of depression during periods of high infection rates and restrictions on social activity; but that there was no effect on anxiety and loneliness.

#### The Negative Effects of Social Cohesion

Some authors offered commentary on the opposites of social cohesion, or its less-desirable effects. These include social cohesion or elements of it as a driver of increased infection or death rates. Ransome et al [53], using data collected from across the United States, and Thomas et al [56], in a San Francisco–based simulation, suggested that social cohesion, viewed as collective engagement or frequency of interpersonal connection respectively, may offer a mechanism by which increases in social cohesion may explain ethnic differences in infection rates. Brief commentary was also made by Hangel et al [121], Zimmermann et al [122], and Schneiders et al [46] on how demands made by commitments to the cohesion of a larger social group might come to negatively impact the well-being of those in one’s close circle. One example of this is in the demands for physical distancing to stop the population-level spread of SARS-CoV-2, meaning that some family members experience isolation.

Some works presented commentaries suggesting that social cohesion may operate to produce in-group versus outgroup orientations or sharpen existing ones in populations in the pandemic environment and explored some of the implications [58,80,82,90,98,114]. Comment was made on group constructions that preexisted the pandemic and their boundaries and operation in its context: age [82], ethnicity [62,65,79,82,86,106,130], gender [114], wealth [79,130] and neighbors versus “outsiders” [58,90]. Others suggested that new groups have formed in response to the pandemic and the requirements of the public health response [80,98]. Ergler et al [80] offered an analysis of the procession of groups that have become marked as outsiders because of the Aotearoa-NZ pandemic response, which included political efforts to generate national unity in opposition to the virus. Schuessler et al [98] measured the extent to which compulsory vaccination policy in Denmark brought into being group boundaries that excluded those resisting vaccination from some aspects of social life.

### Changing Social Cohesion During the Pandemic

#### “Increasing” and “Decreasing” Cohesion During the Pandemic

Some commentators suggested that the pandemic conditions facilitated increases in social cohesion in the manner in which they constructed it. These suggestions were based on observations of increases in the provision of social support [52,74,82,109]; the fruitful interaction between pandemic conditions and previous policy efforts to bolster social cohesion at the local level [48,76]; and perceptions of increases in local unity and solidarity [13,43,44,71,94]. For example, Morgan et al [82] conducted in-depth interviews with older people following the first wave of the COVID-19 pandemic in Aotearoa-NZ and found participants feeling a greater sense of belonging in their communities due to the help they received from family and friends during a difficult time, this being experienced in different manners by different ethnic and age groups.

Some (including some of the same authors) also painted the opposite picture: the pandemic environment bringing about a reduction in social cohesion. Such evidence presented includes declining trust in others from the population under investigation [48,60,71], and in politicians and political institutions [43,44,70,71]. Perceptions of declining national unity are also referenced in this respect [13,43,44,70]. Others, as covered above, reference the action of policy responses to the pandemic to create division [67,80,98]; and the occasional failure of national policy approaches to cater to and include all groups [82,108]. Also raised in this respect is the sometimes polarizing effects of pandemic politics and public health responses and the interplay thereof with social cohesion, adherence to public health measures, and population health outcomes [98,102,123,126]. Three contributions, characterizing social cohesion as mutual support and trust between neighbors, reported no change [57,79,105].

Several works presented social cohesion and division as antithetical and offered broad commentaries on the operation of the latter [13,43,44,115,126,129]. For example, Bisiada [126], in a theoretical discussion of social life in Germany and Spain over the course of the pandemic, countered the argument that social divisions have been primarily ideological in nature and presented another, that socioeconomics have been the major axis. Abrams et al [43,44] presented data to show that the United Kingdom’s experiences of the COVID-19 pandemic have been marked by increasing perceptions of unity at the local level but of disunity at the national level.

#### “Rally Round the Flag” and Its Diminishing Returns

An observation that is made frequently across the literature, especially in works that have engaged in longitudinal research or ongoing analysis, is that there was a moment of increased cohesion in the early stages of the COVID-19 pandemic, which gave way to a return to form—or worse—toward the end of 2020. This was characterized by the British Academy [13] as a “rally round the flag” effect. This effect has been described occurring in relation to a range of different constructions of social cohesion and their indicators: interpersonal interaction, social support, and feelings of togetherness [13,52,87]; trust in “most people” [66], those in one’s neighborhood [42] and government [13,43,96]; political polarization [102]; feelings of national and local unity [13,42-44]; commitment to a common goal [101]; and collaboration between nations [118]. The British Academy’s [13] UK-based narrative suggested preexisting division and declining cohesion in the years before the COVID-19 pandemic, followed by a “coming together” during the first wave, in which people were brought to trust each other more, have increased perceptions of local and national unity, identify more closely with their communities, and offer both emotional and practical support to others. The authors suggested that this moment had come to an end by September 2020, at which point almost all indicators returned to prepandemic levels. Perceptions of local unity in some cases remained higher, and trust in government declined to still lower levels, the latter effect being most pronounced in socioeconomically deprived communities and “key” workers, such as those used in frontline social care, who were concentrated in these lower socioeconomic strata and who experienced the greatest exposure to risk of contracting the virus.

#### The Effects of Policy

A smaller body of work looked into the effects of policy on social cohesion in pandemic conditions. There have been those already mentioned who held vaccination policies [98] and a framing of collective action against the virus [80] as responsible for driving division. Others pointed to the greater levels of social cohesion experienced by nations that focused policy toward fostering it in their pandemic response—including those emphasizing solidarity and agency [114]; “we-ness,” that is, feelings of unity [115]; and the swift rollout of generous social protections [116]. Contrary to this, Strupat [99], in an analysis of the Kenyan response, considered that the burden placed on the nation by the COVID-19 pandemic was too great for the welfare approach overseen by the government to have any real effect. In the study by Di Giulio et al [108], the extended critique of Bolsonaro and the central government’s role in the pandemic response in Brazil shows it to have damaged a version of social cohesion centered on political trust and civic engagement, and, in turn, to have led to worse population health and widened inequality. A further set of commentaries, all from the early stages of the COVID-19 pandemic, offered analyses and advice for policy authors toward prioritizing social cohesion in the pandemic response [50,117,124,125].

#### Changing Interpersonal Relationships

A significant thread in the literature, involving those constructions of social cohesion where interpersonal relationships are at the center, is analysis of how changes in relationships brought about by the COVID-19 pandemic have affected social cohesion. Such accounts include the suggestions that frequency of contact and quality of relationships with people from outside the family circle have diminished [41,43,44,68,84,120]; and that bonding with those in one’s close environment has strengthened [42-44,46,82,120]. Other interesting observations on the changing nature of interpersonal relationships includes their becoming politically polarized or depolarized over time [46,102]; and the manners in which the requirements of remaining physically distant can function in cultures of communal sociality [88,112].

A common suggestion is that the conditions of the COVID-19 pandemic have produced a fundamental reformation of the manners and practices by which social relationships are enacted—and therefore a change in a cohesion that is reliant on these. These range from, most simply, the observation that the novel social conditions of the pandemic may bring new conditions for the construction of personal identity, group identity, and thus group interactions [134], that the extent of disruption to established practices has required a conscious and intentional reformation of relationships [46], to the idea that times of crisis bring the requirement for specific sets of practices of interaction [69,73,97]. More specific observations include those on details of the large-scale shift of interaction to remote communication technologies [52,74]; the use of outdoor singing as a communal negotiation of grief and solidarity [100,133]; and signing up to volunteer programs as an expression of commitment to the collective and a means of engaging in meaningful interpersonal interaction [45,47,68]. There are numerous commentaries dealing with the changing practices of interaction of different groups, including the changing construction of oldness in relation to older people’s increased vulnerability during the pandemic and how this has affected intergenerational relations [74,82,113]; the “closing ranks” of transgender people in expectation of heightened oppression [97]; increasing victimization experienced by ethnic minorities [43]; the increase in disconnection and isolation felt by key workers [45]; and the disproportionate decline in quality and frequency of social relations felt by socioeconomically marginalized peoples [13,115,116,126] and those of lower formal education levels [68].

Of particular interest due to their novel nature are the accounts that identify the intersection between local culture and pandemic environment to create conditions of possibility for new practices of interaction. Schröder et al [69], upon their aforementioned theoretical model in which cohesion arises in a space of latency from an underlying milieu of social relations, noted that in Germany these practices took different forms in different geo-cultural regions. These authors held the distribution of socioeconomic resources and values to be of special importance. Villalonga-Olives et al [90] described a “bounded solidarity” on the island of Menorca in which there was an increase in solidarity and support in the communities of the island but a tension between mistrust of outsiders as potential carriers of disease and the reliance on the same outsiders to bring in capital to maintain islanders’ financial well-being.

### Critique

The literature on social cohesion and the COVID-19 pandemic contains numerous weaknesses. A large proportion of these limitations are a function of the already well-documented issues with the social cohesion concept. The broad diversity of definitions and constructions of social cohesion have long been the subject of discussion and appear to be no closer to a resolution [7,8,12]. In respect of the current work, this means that there is such a spread of versions of social cohesion in circulation that its explanatory power is weakened and it is difficult to make stable comparisons across the body of knowledge. In general, the observation by Bernard [17] remains apt: social cohesion appears as a “quasi-concept,” amenable to the imprinting of any political-ideological or discipline-bound framework within which those using it are working.

The presence of such diversity within social cohesion and its value-laden construction would logically require explicit theoretical engagement and clear justification of choice for the version of the concept being deployed. With some notable exceptions [69,82], this work is not—beyond an acknowledgment of diversity—undertaken with detailed attention in the literature reviewed. As such, social cohesion does not carry as much explanatory strength in analyses of the conditions of the pandemic as it might: a model built without theoretical justification leaves its conclusions open to question and becomes open to such criticisms as that from Schröder et al [69]: that the concept is in danger of becoming an “empty signifier.” This issue is compounded by the use of measures that do not appear to be a good operationalization of the definition of social cohesion provided. It is a frequent occurrence in the literature that the diversity across constructions is acknowledged, a brief summary given, and a particular definition settled upon, following which a measure is chosen for its previous validation or its ease of administration rather than its reflection of the construction of social cohesion offered.

In this body of literature, social cohesion is, as mentioned, situated among a group of concepts clustered around a set of behaviors, orientations, and situations. Social cohesion is frequently not precisely defined or distinguished from those adjacent concepts. This is especially true in respect of social capital, where the two are often treated as synonymous [72,89] or where social capital is used as a proxy for social cohesion without a detailed justification [40,96]. There is also frequent unacknowledged definitional imprecision across social cohesion and “solidarity” [50,121]; engagement with democratic processes [123]; the social contract [66,116]; and activity in the civic space [103,104]. Where these concepts are not defined and differentiated with care and clarity, analyses become unclear as to the networks of cause and effect being invoked, and, again, explanatory power is impeded.

It is possible that a large portion of the imprecision across social cohesion, social capital, and civic engagement is a consequence of the broad influence of Putnam’s [26,28] work on the field and the use of this and other models derived from it. Putnam’s framework, nominally of social capital, incorporates several diverse elements. It situates social cohesion in part as a product of interpersonal connections and individuals’ access to resources (finance, support, etc); a notion present in other constructions of social capital [24,25]. However, it also measures political and civic engagement and uses whole groups as the units of analysis—practices that might sit more comfortably under the social cohesion banner. This may invite conflation of these concepts. The frequent use of Putnam’s framework also means that the body of knowledge focusing on social cohesion during the pandemic is heavily skewed toward a cluster of constructions of social cohesion kept inside its boundaries: at the local or neighborhood level, contingent on its specific set of indicators and its knowledge produced by quantitative inquiry. One common consequence of this is a self-fulfilling deficit orientation by which social cohesion is held to be reliant on face-to-face interpersonal interaction and trust and is measured quantitatively with models designed for normal conditions. However, in the context of stay-at-home and physical distancing requirements, this leads to an inevitable conclusion that there are problems for social cohesion as a result.

Inattention to theoretical justification and the overrepresentation of quantitative models are made more problematic by an overreliance on older self-reported measures of social cohesion that impede the quality of knowledge in the area. Although the complexity and local specificity of social relationships and the unprecedented nature of the pandemic environment are well recognized, the measurement of social cohesion using, for example, Likert scales with ≤5 items—sometimes one alone—is common. This is unlikely to be sufficient to describe the complexity of the relationships across groups and the populations under scrutiny, especially in novel circumstances. One such frequently deployed tool is the scale by Sampson et al [137]. This was devised in 1997 to measure disorder and collective efficacy (over and above social cohesion) in the suburbs of Chicago and measures a small number of perceptions of the neighborhood on a 5-item Likert scale. Aside from the potential problems with validity in the novel pandemic context, the problems with making firm conclusions on self-reported data of this kind are well documented [138]. Even when cultural specificity and the newness of the pandemic environment are explicitly acknowledged, the complexity and diversity of social relations are recognized, and the ongoing issues with the social cohesion concept engaged with, some still fall back on such limited manners of measurement.

At the other end of the spectrum, there are some models that appear to be so large and all-encompassing that social cohesion becomes the basic determinant of almost all psychosocial life and culture. For example, the construction by Godara et al [135] includes social connections (capital), interaction, inclusion, civic engagement, identity, social structures, norms and values, loyalty, solidarity, human rights, trust, conflict management, equality, and order; across micro-, meso-, and macro-levels; involves both structures and groups of various sizes; and includes vertical and horizontal relationships. Scholarship of this kind creates a version of social cohesion that becomes deterministic, mechanistic, and inattentive to local difference, while at the same time being too large to make work within the limits of the capacities of knowledge-production apparatus. Its broadness also, again, weakens its explanatory power. There may be a sweet spot between reductionism, expansionism, and the particular that few contributing to this body of knowledge have managed to achieve, although there are some notable examples, predominantly using qualitative or mixed methods [13,43,82,90].

Two further problems with the literature reviewed here are also noted by commentators on the social cohesion literature more generally. The first, in relation to the quantitative work, and described previously by Janmaat [139] and Green and Janmaat [11], is that the indicators contained within commonly deployed constructions of cohesion almost always do not covary [75]. This calls into question the validity of the construction of a concept that aims to provide a single coherent explanation—or independent variable—for social life. The second is that there is some ethical concern with works [53,56] that apply closed-ended quantitative approaches to identify ways in which social cohesion may be driving higher rates of sickness and death from COVID-19 in marginalized populations and minorities. As has been pointed out elsewhere [140], this comes with the danger of blaming the victims of structural violence for its effects.

A final comment here relates to the quality of data regarding COVID-19 outcomes. Such was the speed at which the pandemic achieved great size, the data collection infrastructures were overwhelmed, and the quality of data describing its effects is not currently of a quality most would hope to have. This means that commentaries on effects on population health outcomes, especially those relying on quantitative data, should be treated with caution until better data are produced. This effect is most prominent in those commentaries undertaken early in the pandemic when the quality of data was at its worst. Furthermore, a number of those accounts authored in the early stages of the pandemic may have produced different conclusions if they were undertaken with a longer period of experience, evidence, and context from which to draw.

## Discussion

### Principal Findings

It is valuable to understand the ways social relationships have influenced and have been influenced by the COVID-19 pandemic. This work reviewed the body of literature that has made use of the social cohesion concept in this endeavor. A range of different constructions of social cohesion were found and can be categorized into the following broad groups:

1. Those considering it a product of interpersonal relationships
2. Those claiming a reliance on sameness
3. Social cohesion as collective action or acting for the benefit of the collective
4. The accumulation of individual subjective perceptions or emotions relating to togetherness
5. The operation of structures of governance
6. Locally or culturally specific arrangements
7. Hybrid models

Some commentaries center on the effects of social cohesion on other objects or processes of interest. In these, the following broad themes are present:

1. Cohesive groups or societies are generally said to see lower burdens of ill-health during the pandemic, depending on how social cohesion is constructed and the population under study.
2. Cohesive groups or societies are generally said to engage in better health-related practices in the context of COVID-19, though there are some tensions identified between the requirements of the larger collective and smaller groups.
3. Social cohesion is said to be a resource for resilience, emotional well-being, and protection against clinically diagnosable mental states during the pandemic.
4. There are indications of an emergence of novel social groupings in relation to the demands of the pandemic and related policy and evidence of in-group–outgroup dynamics.

Some work describes social cohesion itself during the pandemic. In such scholarship, the following broad themes are present:

1. Changes to social cohesion claimed during the pandemic depend on the way it is constructed and the groups under investigation. Changes are distributed and experienced unequally.
2. There was a “rally round the flag” moment early in the pandemic where many populations exhibited higher social cohesion by many different appraisals. This gave way to a return to type—or worse—toward the end of 2020.
3. Government policy before and during the pandemic has been of real importance to the operation of social cohesion ongoing.
4. There have been significant and fundamental changes to the practices around interpersonal relationships. These changes have not been distributed equally across populations.

Problems identified in the literature by and large reflect the wider and well-documented issues with the social cohesion concept and the diversity of forms it takes [12,17,36]. This makes comparison across the literature and the development of coherent thematic structures somewhat problematic. There is an overreliance on long-established quantitative tools of measurement, which are likely not appropriate for such a new and complex situation. One of the major issues in this respect is the reduction of a complex and diverse arrangement of culturally bound relationships to a limited number of closed-ended measures.

Much of what has been reported here aligns with previous relevant scholarship. Interrelationships between social cohesion and health have been studied extensively, usually suggesting a positive association and a health-promoting effect [29,141]. The importance of social cohesion in preparing for and responding to times of difficulty effectively has also been the topic of extensive scholarship, including violent conflict [142], significant social change [143], natural disasters [144,145] and pandemics [146,147]. Moreover, the transmission of social cohesion to individual psychological and emotional resilience against distress is well studied [148]. The situation-specific forms of bonding, collective action, and support in response to moments of increased need (the “rally round the flag” effect [13]) have also been studied previously under the banner of social cohesion (eg “emergent social cohesion” [149]), such that scholars early in the COVID-19 pandemic engaged in hurried efforts to set out how this effect might be promoted and harnessed [150].

However, new knowledge has been produced by this literature. Obviously, some of this is in relation to COVID-19, which is, of course, itself new, but there has also been novel insight and development of existing ideas on how social cohesion may be conceptualized, its operation, and important considerations therein. A good portion of the more notable insights are raised by qualitative work, which is well-represented in the body of work reviewed here and which is untypical of the social cohesion scholarship in general. One of the more significant directions taken up by the literature reviewed is the acknowledgment of a diversity of potentialities for social cohesion. These included the new arrangements of identities, group membership, relationships, and manners of interaction demanded by the pandemic environment and policy responses [46,47,66,134]. Positions maintaining that distinct locally and culturally bound arrangements of cohesion exist, were also forwarded, taking up Green and Janmaat’s [11] somewhat neglected invitation to investigate the idea using a particularist lens [69,90,103]. Commentaries also extended the understanding of the temporal nature of certain expressions of cohesion [13,43,116].

Another area of interest where significant new ground was broken was the place of information and communication technology in the functioning of social relationships and therefore social cohesion, an area identified as neglected by Bayliss et al [9] in their 2019 review. Commentaries highlighted the importance of communications technology (and, of course, having possession of it and necessary skills to make use of it) for maintaining relationships over distance and therefore for the strengthening of cohesion [52,82]; and thus, by implication calling into question the usefulness of constructions of the concept that rely heavily on the occurrence of face-to-face interaction. In addition, scholarship drew attention to the technologically mediated processes of in and out group formation and political polarization [98,102,126].

This review has several limitations. First, although as broad a net as possible was cast intentionally, the knowledge being produced is bounded by the limits of the search terms deployed. There are, for example, relevant literatures dealing with social capital (without mention of social cohesion), communitas, “tight and loose” cultures, and more that are not captured by the singular focus on social cohesion. Second, there are likely relevant works that were not held by any of the 3 libraries searched here and which have thus been omitted. Third, as has been suggested in the findings section, during the period from which literature was obtained the situation and knowledge on it developed—and in many cases misunderstandings and errors were corrected—at a great pace. This means that those published earlier in the pandemic did not have the benefit of the developments, context, corrections, and hindsight that those published later did. This means that the knowledge being produced here is, at least in part, also subject to this issue. Fourth, related to the third, data quality relating to COVID-19 (especially on morbidity and mortality and, again, especially from early in the pandemic) is still not of the standard one would wish to have to make robust claims. Finally, the great diversity of forms and manners in which social cohesion has been conceptualized, constructed, and measured means that it is frequently difficult and problematic to make broad and sweeping statements on the body of literature. Indeed, as is common across social cohesion literatures, much of the scholarship uses similar nomenclature but is commenting upon and measuring a surprisingly diverse range of phenomena, experiences, and subjectivities.

### Future Work

Overall, there is a need for work in this field to use more care and precision when deploying the social cohesion concept. Perhaps the single most compelling piece of evidence from the current review to support this notion is the large number of papers returned that were excluded due to their neglect to offer a definition of the term or any real detail on what it might add to the analysis. It is introduced all too frequently as if there were consensus as to its conceptual stability and as a public good whose benefits are self-evident—and as such without need for any explication. As has been demonstrated by this review and a long lineage of critical work, there is no such consensus, stability, or grounds for presumption of goodness. It appears that, in the face of the uncertainty and imprecision that have long plagued the concept, any scholarship seeking to make use of it must necessarily undertake important groundwork if it is to be of real value to the body of knowledge.

There are a number of areas where such care and precision might be focused. First, and most importantly, there is an immediate need for engagement with and development of the theory underpinning the range of constructions of social cohesion to sharpen them and make them more useful—both for understanding the pandemic and other aspects of social life. There are some promising beginnings offered by those, such as Schröder et al [69], which engage in robust theoretical justification for their choices in their process of model construction, allow for flexibility of group boundaries, and seek to capture important context-specific features of social life. Such works may be built upon and put to use in improving the concept, its components, and ways of measurement toward understanding the social world. Theoretical work currently appears especially necessary for establishing the robust connections between constructions of social cohesion and the variables chosen for its representation, which are currently largely sorely lacking. Such work will help both to sharpen the concept and end the overreliance on older and more limited measures. This will also necessarily involve considered and explicit disambiguation of the cluster of concepts that have thus far been confused—especially social cohesion and social capital.

Following this, there is a need for more qualitative research to generate new knowledge on the ways social cohesion may be understood to operate in the context of COVID-19 and beyond. Work, such as that from Morgan et al [82], appears to push at the boundaries and demand evolution of the older model of social cohesion selected for use. This is a demonstration of the usefulness of detailed and in-depth qualitative work for understanding the unprecedented social conditions and relationships operating during the pandemic and of the opportunity to develop the existing quantitative frameworks toward greater depth of understanding. Scholarship in both arenas will assist both in improving the social cohesion concept and the social cohesion scholarship on the COVID-19 pandemic in relation to contextual specificity. The importance of this line of inquiry has been noted by Green and Janmaat [11] for social cohesion generally and by Schröder et al [69] on the pandemic environment, but work of this nature remains thin on the ground. Work to explore subjective perceptions of togetherness, the reorganization of priorities and practices taking place during the pandemic, and the effects of swift and radical new policy directions would all be of value.

The literature contains an overrepresentation of analyses conducted at the neighborhood level and, relatedly, a preponderance of constructions of social cohesion that may more comfortably be called social capital. This is true of both the COVID-19–related scholarship and the wider social cohesion field [140]. More work is needed to understand the operation of societies at the national level. The ongoing project conducted by Abrams et al [42-45] in the United Kingdom offers an excellent example of the value of such scholarship. There is also room for more (and more detailed) analysis at the small-group level under the social cohesion banner; for example, what are the subjective mechanics of togetherness and identification with the group in the pandemic environment? The operation of information and communication technology in respect of social cohesion also represents a promising avenue for future research offered by the work reviewed: in the pandemic’s requirement for physical distance, what does the replacement of interpersonal interaction with technologically mediated modes mean for the nature of togetherness? This may also provide interesting and useful insights around the notion of cohesive transnational groups and populations connected by such technologies beyond the pandemic environment.

## Abbreviations

Aotearoa-NZ: Aotearoa–New Zealand
